# HIF2a inhibitors for the treatment of VHL disease

**DOI:** 10.18632/oncotarget.4564

**Published:** 2015-06-25

**Authors:** Ana Martins Metelo, Haley Noonan, Othon Iliopoulos

**Affiliations:** Massachusetts General Hospital Cancer Center and Harvard Medical School, Charlestown, MA, USA

**Keywords:** hypoxia, VHL, HIF2a inhibitors, renal cancer

Patients with von Hippel-Lindau (VHL) disease possess a germline mutation in the VHL tumor suppressor gene that confers a life-time risk of developing renal cell carcinomas (RCC), central nervous system hemangioblastomas (HB), pheochromocytomas, pancreatic neuroendocrine tumors, papillary cystadenomas and erythrocytosis [1]. The VHL protein targets the Hypoxia Inducible Factors 1 and 2a (HIF1a and HIF2a) for proteasomal degradation in cells exposed to a normal range of oxygen concentration. However, low oxygen concentration (hypoxia) or loss-of-VHL function lead to HIF1a/2a stabilization and transactivation of HIF-target genes. HIF1a/2a are transcription factors targeting genes such as vascular endothelial factor (VEGF), transforming growth factor (TGF), erythropoietin (EPO), erythropoietin receptor (EPOR), transferrin, and angiopoietin 1. Collectively, the expression of HIF1a/2a target genes contributes to oncogenic processes such as angiogenesis, erythropoiesis, reprogramming of metabolism, cell proliferation, and metastasis [1]. HIF1a and HIF2a are paralogs expressed in most human epithelial cells and possess both overlapping and distinct functions [2]. For example, in RCC, it is known that HIF2a acts as an oncogene, while HIF1a is a tumor suppressor gene [3].

There are currently no drugs available to treat VHL disease. VHL patients develop multiple tumors over a lifetime that require repeated surgeries. Not only can such surgeries for serially appearing lesions result in damaged renal or brain parenchyma, but oftentimes they are not feasible due to the location of the HB [4]. Therefore, pharmacological inhibition of HIF2a would be an ideal therapeutic strategy in the treatment of VHL disease and HIF2a-driven tumors. We review here our recent work and present for the first time evidence that small molecule HIF2a inhibitors, developed by the Iliopoulos Laboratory at Massachusetts General Hospital and Harvard Medical School, target HIF2a in vivo, using a vertebrate animal model of human VHL disease.

We previously identified small molecule HIF2a inhibitors via a mammalian cell-based reporter screen of HIF2a activity [5]. These inhibitors operate by enhancing the binding of iron regulatory protein 1 (IRP1) to an iron regulatory element (IRE) in the 5′-UTR of HIF2a, but not HIF1a mRNA, thereby specifically repressing HIF2a translation. In our recent study, published in Journal of Clinical Investigation (Metelo AM et al., JCI 2015;125 (5):1987-97), we provide evidence that the HIF2a inhibitor, lead compound 76, can inhibit the zebrafish orthologs of human HIF2a and ameliorates significantly the phenotypic abnormalities of the vhl−/− embryos. This work indicates that pharmacological inhibition of HIF2a is sufficient to treat VHL-disease related abnormalities. In addition, it provides strong rational for further preclinical development of these HIF2a inhibitors.

Zebrafish possess two orthologs of human HIF2a, called epas1a and epas1b, as well as two orthologs of human HIF1a, hif1aa and hif1ab. We previously showed that only human HIF2a contains a 5′-UTR with a functional IRE, unlike HIF1a, and consequently, compound 76 is specific for HIF2a and does not suppress HIF1a translation in mammalian cells [5]. We proved that the same is true for the 5′-UTR of zebrafish Hif2a orthologs, epas1a and epas1b.

To test whether compound 76 has the ability to repress epas1a and epas1b activity in vivo we challenged wild type zebrafish embryos with a chemical hypoxia mimetic, DMOG. Treatment of animals with DMOG results in stabilization of all zebrafish orthologs of human HIF1a/2a and robust upregulation of their target genes (phd3, epo, and vegfab). Compound 76 suppressed the expression of hypoxia-target genes in zebrafish. Morpholino knockdown experiments strongly suggest that hypoxic expression of epo and vegf is primarily controlled by the Hif2a paralogs, epas1a and epas1b. Suppression of epas1a and epas1b by compound 76 was biologically impactful; compound 76 significantly suppressed the epo-driven erythrocytosis and angiogenesis that followed exposure of embryos to DMOG.

In the process of quantifying the effect of inhibitor 76 we developed, in collaboration with the Carpenter Laboratory at the Broad Institute, a computerized imagebased assay that allows the quantification of angiogenesis and erythropoiesis in zebrafish embryos. This novel method can now be applied to high-throughput screens for the identification of compounds that regulate angiogenesis and erythropoiesis in vivo.

Zebrafish embryos, homozygous for vhl loss-offunction mutations (vhl−/− embryos), resemble human VHL disease and develop epo-driven erythrocytosis, complex blood vessel networks in the brain and retina reminiscent of HB, increased proliferation of their liver and kidney that is reflective of VHL-associated tumor biology, and cardiomegaly with decreased cardiac contractility [6]. We used vhl−/− embryos to test the in vivo effect of the HIF2a inhibitors that we identified. We found that compound 76 significantly suppresses the expression of epas1a/1b-target genes (phd3, epo, transferrin, vegfab, angiopoietin 1, and tgfa) in vhl−/− mutant embryos. The effect of the inhibitor was not merely biochemical; compound 76 suppressed the epo-driven erythrocytosis as well as the abnormal vascular proliferation seen in the brain and trunk characterizing the vhl−/− embryos. In addition, compound 76 promoted erythroid differentiation and decreased the number of early erythroid progenitors circulating in the peripheral blood. The number of erythroid progenitors is characteristically increased in vhl−/− embryos, possibly as a direct effect of epas1a/1b on erythroid maturation. Finally, we found that the HIF2a inhibitor compound 76 significantly improved cardiac contractility among the vhl−/− embryos and enhanced their viability.

In conclusion, our work presents an example of pharmacological treatment of VHL disease related abnormalities in a vertebrate animal model. HIF2a inhibitor 76, a lead compound, exhibits promising in vivo activity as it can partially reverse the VHL phenotype in zebrafish. These observations provide a strong rationale for optimization of compound 76 via medicinal chemistry to develop derivatives suitable for preclinical and clinical testing. The VHL gene is inactivated in over 90% of sporadic RCC tumors [7], rendering RCC a model disease for HIF2a inactivation. Specific HIF2ainhibitors can be used not only to treat VHL disease but the majority of these sporadic RCC as well. In addition, there is compelling preclinical and clinical evidence for the contribution of HIF2a expression in several human malignancies (such as glioblastoma, lung, colon, ovarian and prostate cancer).
